# Late HIV Diagnosis and Determinants of Progression to AIDS or Death after HIV Diagnosis among Injection Drug Users, 33 US States, 1996–2004

**DOI:** 10.1371/journal.pone.0004445

**Published:** 2009-02-13

**Authors:** Anna Grigoryan, H. Irene Hall, Tonji Durant, Xiangming Wei

**Affiliations:** 1 Division of HIV/AIDS Prevention, Centers for Disease Control and Prevention (CDC), Atlanta, Georgia, United States of America; 2 McKing Consulting Corporation, Atlanta, Georgia, United States of America; University of Nebraska, United States of America

## Abstract

**Background:**

The timeliness of HIV diagnosis and the initiation of antiretroviral treatment are major determinants of survival for HIV-infected people. Injection drug users (IDUs) are less likely than persons in other transmission categories to seek early HIV counseling, testing, and treatment. Our objective was to estimate the proportion of IDUs with a late HIV diagnosis (AIDS diagnosis within 12 months of HIV diagnosis) and determine the factors associated with disease progression after HIV diagnosis.

**Methodology/Principal Findings:**

Using data from 33 states with confidential name-based HIV reporting, we determined the proportion of IDUs aged ≥13 years who received a late HIV diagnosis during 1996–2004. We used standardized Kaplan-Meier survival methods to determine differences in time of progression from HIV to AIDS and death, by race/ethnicity, sex, age group, CD4^+^ T-cell count, metropolitan residence, and diagnosis year. We compared the survival of IDUs with the survival of persons in other transmission categories. During 1996–2004, 42.2% (11,635) of 27,572 IDUs were diagnosed late. For IDUs, the risk for progression from HIV to AIDS 3 years after HIV diagnosis was greater for nonwhites, males and older persons. Three-year survival after HIV diagnosis was lower for IDU males (87.3%, 95% confidence interval (CI), 87.1–87.4) compared with males exposed through male-to-male sexual contact (91.6%, 95% CI, 91.6–91.7) and males exposed through high-risk heterosexual contact (HRHC) (91.9%, 95% CI, 91.8–91.9). Survival was also lower for IDU females (89.5%, 95% CI, 89.4–89.6) compared to HRHC females (93.3%, 95% CI, 93.3–93.4).

**Conclusions/Significance:**

A substantial proportion of IDUs living with HIV received their HIV diagnosis late. To improve survival of IDUs, HIV prevention efforts must ensure early access to HIV testing and care, as well as encourage adherence to antiretroviral treatment to slow disease progression.

## Introduction

Although mortality has decreased significantly and the prognosis for HIV patients has improved since the introduction of highly active antiretroviral therapy (HAART) [1]–[3], the survival of HIV-infected injection drug users (IDUs) may not have improved as much as for persons in other transmission categories. The timeliness of HIV diagnosis and the initiation of HAART are major determinants of survival for HIV-infected people [4]. There is growing evidence that IDUs not only have suboptimal access and adherence to HAART [5], [6] but also tend to begin antiretroviral treatment at a more advanced stage of infection [5], [7]. There are significant public health benefits of early diagnosis and treatment of HIV infection among IDUs. Reports show that IDUs, who know that they are infected with HIV, are more likely to protect their partners from infection reducing further the spread of the virus [8], [9]. An analysis of US population–based data (including IDUs) showed that the survival of IDUs after HIV diagnosis was shorter than that of persons in other transmission categories [10]. Despite earlier research, no findings on late testing and survival data specific to subpopulations of HIV-infected IDUs have been published. We wished to determine whether demographic factors such as race/ethnicity, sex, and age affect prognosis in the HAART era. Therefore, using data from the national HIV/AIDS surveillance system of the Centers for Disease Control and Prevention (CDC), we examined the proportions of subpopulations of IDUs who received a late HIV diagnosis and the determinants of disease progression and survival. Understanding the problem of late HIV diagnosis and determinants of survival for IDUs is important to the development and implementation of HIV prevention efforts.

## Methods

In 1994, CDC implemented a uniform system for national surveillance of HIV infection integrated with AIDS surveillance, and 25 states with confidential name-based HIV infection reporting started submitting case reports to the CDC. Over time, additional areas implemented confidential name-based HIV infection reporting and started submitting data to CDC. For our analyses, data were available from 33 states (Alabama, Alaska, Arizona, Arkansas, Colorado, Florida, Idaho, Indiana, Iowa, Kansas, Louisiana, Michigan, Minnesota, Mississippi, Missouri, Nebraska, Nevada, New Jersey, New Mexico, New York, North Carolina, North Dakota, Ohio, Oklahoma, South Carolina, South Dakota, Tennessee, Texas, Utah, Virginia, West Virginia, Wisconsin, and Wyoming). We analyzed cases in adults and adolescents, aged 13 years and older, for whom HIV diagnosis was made during 1996–2004 and who were reported to CDC through June 2007 from the 33 states. Persons with HIV infection were defined as those who had received a diagnosis of HIV with or without a diagnosis of AIDS at the time of HIV diagnosis. HIV diagnosis was considered late if an AIDS diagnosis was made <12 months after the HIV diagnosis [11]. HIV diagnosis was considered concurrent when AIDS diagnosis was made at or within the same calendar month of HIV diagnosis. AIDS is defined by a CD4^+^ T-cell count <200 cells/µL or by an AIDS-defining illness.

### Progression from HIV Diagnosis to AIDS

To understand patterns in diseases progression, we examined the time from the diagnosis of HIV infection to the time of diagnosis of AIDS among the adolescent and adult HIV-infected IDUs who received a diagnosis of HIV infection during 1996–2004. First, we determined the proportion of IDUs whose diagnosis of AIDS was made within 12 months after HIV diagnosis; then we compared the proportion of IDUs with the proportions of persons in other transmission categories (chi-square test, *P*<.05). We used the hierarchical transmission categories of male-to-male sexual contact, injection drug use, male-to-male sexual contact and injection drug use; and high-risk heterosexual contact (heterosexual contact with a person known to have, or to be at high risk for, HIV infection).

We used a standardized Kaplan-Meier survival method [12] to determine the time from a diagnosis of HIV infection to a diagnosis of AIDS among IDUs, by race/ethnicity, sex, age group, metropolitan residence, and diagnosis year. Cases of HIV were followed up for diagnosis of AIDS through December 31, 2005 (censor date), for AIDS diagnoses reported through June 30, 2007 (allowing 2.5 years for a diagnosis of AIDS to be reported). For individuals who were event free (no AIDS diagnosis reported) at the censor date, it was assumed that the disease had not progressed to AIDS. Data on persons who died during the follow-up period were censored at date of death. The completeness of AIDS and HIV (not AIDS) case reporting was estimated at more than 85% [11]. We calculated the proportion of IDUs whose infection did not progress to AIDS. Race/ethnicity was categorized as white, black, Hispanic, Asian/Pacific Islander, and American Indian/Alaska Native. The categories for age were 13–24, 25–34, 35–44, 45–54, and 55 years and older. The categories for metropolitan residence were metropolitan statistical areas of <50,000, 50,000 to 499,999, 500,000 to 2,499,999 and >2.5 million. Estimates were adjusted for all other factors in our analyses. The date of diagnosis was missing or incomplete for 413 of 27,985 (1.5%) HIV-infected IDUs; the data for these 413 persons were excluded from analyses.

### Survival after HIV Diagnosis

Using data from 33 states, we determined the survival of adolescents and adults for whom HIV infection was diagnosed during 1996–2004. Cases were followed up through December 31, 2005 (i.e., censored), for deaths reported through June 30, 2007 (allowing 2.5 years for deaths to have been reported). Individuals without a death report at censoring date were assumed to be alive.

First, we determined survival among IDUs and then compared their survival with the survival of persons in other transmission categories. To determine the factors associated with survival among IDUs, we stratified IDUs by race/ethnicity, sex, age group, CD4^+^ T-cell count, concurrent diagnosis of HIV and AIDS (this variable measures severity of disease at diagnosis), metropolitan residence, and diagnosis year. The categories for CD4^+^ T-cell count at HIV diagnosis (first CD4^+^ T-cell count reported within 6 months after diagnosis) were: <50, 50–99, 100–199, and ≥200/µL (categories used in earlier prognostic models [13]); CD4^+^ T-cell count was missing for 68.0% of cases. We present the 1-year and 3-year survival estimates by using directly standardized Kaplan-Meier techniques [12]. Significant differences between groups were assessed by determining whether the 95% confidence intervals overlapped. Of all 236,021 HIV cases diagnosed during 1996–2004, 3,334 (1.4%) and of 27,478 HIV-infected IDUs, 451 (1.6%) respectively did not have complete information on date of diagnosis, and were excluded from analyses.

## Results

### Late HIV Diagnosis and Progression from HIV Diagnosis to AIDS

Of the 27,572 cases of HIV infection diagnosed among IDUs in the 33 states during 1996–2004, HIV infection in 57.8% did not progress to AIDS within 12 months after HIV diagnosis (Table 1, see column 3). In other words, in 42.2% of IDUs, HIV infection progressed to AIDS within 12 months, and these IDUs were therefore considered to have received a late diagnosis. A significantly larger proportion of IDUs received a late diagnosis, compared with persons in other transmission categories: men who have sex with men [MSM], 39.8%; MSM who were also IDUs, 38.6%; and heterosexual adults at high risk, 36.6% (*P*<.*0001*; data not shown).

**Table 1:** Percentage of IDUs with HIV diagnosis who did not progress to AIDS in 1996–2004, 33 U.S. States.

Race/ethnicity	No	%	Percent without AIDS 1 year after HIV diagnosis		Percent without AIDS 3 years after HIV diagnosis	
			%	95% CI	%	95% CI
White	7,229	26.2	57.0	56.5 , 57.5	49.9	49.6 , 50.2
Black	15,616	56.6	57.7	57.1 , 58.2	48.9	48.5 , 49.2
Hispanic	4,456	16.2	57.3	56.8 , 57.8	48.3	47.8 , 48.8
Asian/Pacific Islander	57	0.2	47.3	—	39.3	—
American Indian/Alaska Native	214	0.8	49.9	49.7 , 50.0	41.4	—
**Sex**						
Male	17,927	65.0	54.5	54.1 , 55.0	46.2	45.9 , 46.6
Female	9,645	35.0	63.9	63.4 , 64.4	54.9	54.5 , 55.2
**Age (years)**						
13–24	1,339	4.9	79.8	79.5 , 80.1	71.7	—
25–34	5,942	21.6	64.8	64.1 , 65.5	55.9	55.5 , 56.4
35–44	11,718	42.5	55.9	55.2 , 56.5	47.3	46.9 , 47.6
45–54	7,148	25.9	51.7	51.1 , 52.3	43.2	42.7 , 43.7
≥55	1,425	5.2	46.6	45.9 , 47.3	38.2	37.6 , 38.8
**Metropolitan residence**						
<50,000	2,502	9.1	58.1	57.1 , 59.0	49.8	49.5 , 50.1
50,000–499,999	4,278	15.5	57.6	57.1 , 58.2	50.5	50.0 , 50.9
500,000–2,499,999	14,096	51.1	57.4	56.9 , 58.0	49.0	48.7 , 49.4
≥2.5 million	6,696	24.3	57.5	56.8 , 58.2	48.0	47.7 , 48.3
**Diagnosis year**						
1996	3,315	12.0	54.1	53.2 , 55.0	46.2	45.6 , 46.8
1997	2,676	9.7	54.4	53.3 , 55.5	46.6	45.8 , 47.4
1998	2,221	8.1	55.1	54.2 , 56.0	48.5	47.9 , 49.2
1999	2,834	10.3	56.3	55.2 , 57.4	47.3	46.8 , 47.8
2000	3,289	11.9	56.9	55.9 , 57.8	48.2	47.6 , 48.8
2001	4,430	16.1	59.4	58.4 , 60.4	51.0	50.5 , 51.5
2002	3,449	12.5	59.9	58.9 , 61.0	50.7	49.8 , 51.7
2003	2,872	10.4	59.2	57.9 , 60.4		
2004	2,486	9.0	56.8	55.6 , 57.9		
**Total**	**27,572**	**100.0**	**57.8**	**57.5 , 58.2**	**49.3**	**49.1 , 49.6**

Estimates are from standardized Kaplan-Meier analyses, adjusted for all other factors shown in the table. Data reported to CDC through June 2007. Dash indicates data not presented because the variance for the estimate is zero.

Among HIV-infected IDUs, a larger proportion of males (45.5%) than females (36.1%) received a late diagnosis (Table 1). By race/ethnicity, the largest proportion of those with a late diagnosis comprised Asians/Pacific Islanders (52.7%), followed by American Indians/Alaska Natives (50.1%), whites (43.0%), Hispanics (42.7%), and blacks (42.3%). The proportions of persons with a late diagnosis increased with age—from 20.2% of those aged 13–24 to 53.4% of those aged 55 and older.

At 3 years, HIV infection in IDUs was least likely to have progressed to AIDS in whites, compared with blacks, Hispanics, Asians/Pacific Islanders, and American Indians/Alaska Natives (Table 1, column 5). HIV infection did not progress to AIDS in 46.2% of males (95% CI, 45.9–46.6) compared with 54.9% of females (95% CI, 54.5–55.2). AIDS was significantly more likely to develop within 3 years among older IDUs than among younger IDUs. The proportion of IDUs in whom HIV infection did not progress to AIDS did not differ substantially by metropolitan residence. The proportion of IDUs in whom HIV infection did not progress to AIDS was larger in later years (2002 and 2001) than in 1996.

### Survival after HIV Diagnosis

Of 232,685 persons in 33 states who received a diagnosis of HIV infection (with or without concurrent AIDS) during 1996–2004, 27,027 (11.6%) had IDU-associated HIV diagnoses. Fewer IDUs, compared with persons who had other identified risk factors, survived 3 years after HIV diagnosis (Table 2, see column 3 for 1-year and column 5 for 3-year survival). Of 27,027 IDUs in 33 states who received a diagnosis of HIV infection during 1996–2004, a total of 5,896 died by the end of 2005. Twenty-five percent of IDUs were diagnosed with HIV and AIDS at the same time, and among those IDUs 38.2% had very low CD4^+^ T-cell counts at HIV diagnosis (<50 cells/µL) (data not shown). Survival was substantially shorter for IDUs with concurrent diagnosis of HIV and AIDS compared with IDUs who had an HIV diagnosis (not AIDS) only (Table 3). Survival among IDUs did not differ substantially by sex. The analysis of the survival of IDUs by race/ethnicity, at 3 years revealed that smaller proportions of American Indians/Alaska Natives (82.8%) and blacks (85.8%, 95% CI, 85.6–85.9) had survived. Fewer older IDUs survived 1 and 3 years after HIV diagnosis compared with those who received a diagnosis when they were younger. Survival was longer for IDUs with higher CD4^+^ T-cell counts, but this information was missing for 68.0% of patients (most of whom had received a diagnosis of HIV without AIDS). Fewer of the IDUs living in metropolitan areas, compared with nonmetropolitan areas, survived 1 and 3 years after HIV diagnosis. In later years (2002 or 2001), compared with 1996, a slightly larger proportion of IDUs had survived.

**Table 2:** Survival of persons with HIV* diagnosis by transmission category in 1996–2004, 33 U.S. States.

Transmission category	No	%	Probability of surviving 1 year after HIV* diagnosis		Probability of surviving 3 years after HIV* diagnosis	
			%	95% CI	%	95% CI
Male-to-male sexual contact	82,673	35.5	95.2	95.2 , 95.3	91.6	91.6 , 91.7
Injection drug use						
Male	17,528	7.5	93.2	93.1 , 93.4	87.3	87.1 , 87.4
Female	9,498	4.1	95.1	95.0 , 95.3	89.5	89.4 , 89.6
Male-to-male sexual contact and injection drug use	7,616	3.3	95.5	95.5 , 95.6	90.7	90.6 , 90.8
High-risk heterosexual contact						
Male	18,670	8.0	95.7	95.6 , 95.8	91.9	91.8 , 91.9
Female	33,708	14.5	96.8	96.7 , 96.9	93.3	93.3 , 93.4
Unknown/other						
Male	38,837	16.7	92.4	92.3 , 92.5	88.1	88.1 , 88.2
Female	24,155	10.4	94.8	94.7 , 94.9	90.9	90.8 , 91.0
**Total**	**232,685**	**100.0**				

* Diagnosis of HIV infection with or without a concurrent diagnosis of AIDS.

**Table 3:** Survival of IDUs with HIV* diagnosis in 1996–2004, 33 U.S. States.

Race/ethnicity	No	%	Probability of surviving 1 year after HIV* diagnosis		Probability of surviving 3 years after HIV* diagnosis	
			%	95% CI	%	95% CI
White	7,089	26.2	93.3	93.2 , 93.5	87.1	86.9 , 87.3
Black	15,312	56.7	92.8	92.6 , 93.0	85.8	85.6 , 85.9
Hispanic	4,360	16.1	94.3	94.2 , 94.5	88.1	88.0 , 88.3
Asian/Pacific Islander	57	0.2	97.9	—	97.9	—
American Indian/Alaska Native	209	0.8	94.1	—	82.8	—
**Sex**						
Male	17,528	64.9	92.7	92.6 , 92.8	85.9	85.8 , 86.1
Female	9,498	35.1	93.8	93.7 , 93.9	87.1	87.0 , 87.2
**Age (years)**						
13–24	1,332	4.9	98.4	98.3 , 98.5		
25–34	5,893	21.8	95.9	95.8 , 96.0	91.1	91.0 , 91.2
35–44	11,508	42.6	93.4	93.2 , 93.6	86.7	86.5 , 86.9
45–54	6,924	25.6	91.6	91.3 , 91.8	82.3	82.1 , 82.5
≥55	1,370	5.1	84.2	84.0 , 84.4	75.6	75.4 , 75.8
**CD4** ^+^ **T-cell count within 6 months after AIDS diagnosis**						
<50	3,516	13.0	81.6	81.2 , 82.0	69.0	68.7 , 69.4
50–99	1,582	5.9	85.4	85.3 , 85.5	74.8	74.7 , 74.8
100–199	2,900	10.7	93.1	92.8 , 93.4	84.2	84.1 , 84.3
≥200	643	2.4	95.8	95.7 , 95.8	89.2	89.1 , 89.2
Unknown	18,386	68.0	94.1	93.9 , 94.3	88.2	88.0 , 88.3
**AIDS at HIV diagnosis**						
Yes	6,656	24.6	84.9	84.5 , 85.2	75.1	75.0 , 75.2
No	20,371	75.4	94.4	94.2 , 94.5	87.9	87.7 , 88.0
**Metropolitan residence**						
<50,000	2,453	9.1	94.4	94.1 , 94.6	89.5	89.4 , 89.7
50,000–499,999	4,214	15.6	93.6	93.3 , 93.8	87.3	87.1 , 87.5
500,000–2,499,999	13,806	51.1	92.8	92.7 , 93.0	85.9	85.8 , 86.1
≥2.5 million	6,554	24.2	93.3	93.0 , 93.5	86.5	86.4 , 86.7
**Diagnosis year**						
1996	3,254	12.0	92.2	91.8 , 92.6	84.2	83.9 , 84.4
1997	2,629	9.7	93.0	92.7 , 93.4	85.4	85 , 85.8
1998	2,178	8.1	92.6	92.3 , 92.9	85.6	85.2 , 86.0
1999	2,769	10.2	93.2	92.9 , 93.6	86.2	85.9 , 86.5
2000	3,225	11.9	93.3	93.1 , 93.5	86.5	86.2 , 86.8
2001	4,325	16.0	93.4	93.2 , 93.6	87.2	86.9 , 87.5
2002	3,380	12.5	93.8	93.6 , 94.1	87.9	87.6 , 88.2
2003	2,822	10.4	93.9	93.6 , 94.2		
2004	2,444	9.0	93.3	92.9 , 93.7		
**Total**	**27,027**	**100.0**	**92.8**	**92.7 , 92.9**	**85.9**	**85.8 , 86.0**

Estimates are from standardized Kaplan-Meier survival analyses, adjusted for all other factors shown in the table.

* Diagnosis of HIV infection with or without a concurrent diagnosis of AIDS.

Dash indicates data not presented because the variance for the estimate is zero.

## Discussion

Despite the well-known benefits of early detection and the availability of treatment for HIV infection, 42.2% of HIV-infected IDUs received a diagnosis of AIDS within 12 months after receiving a diagnosis of HIV infection. IDUs whose diagnosis is made late in the course of HIV infection may unknowingly transmit infection and once diagnosed may have worse outcomes. In our analysis we found that survival among IDUs was shorter than among other HIV risk groups. The proportion of IDUs who did not progress to AIDS 3 years after HIV infection was lower among men. IDUs with a diagnosis of HIV at older ages or with more severe disease (lower CD4^+^ T-cell count at AIDS diagnosis) had shorter survival.

Our results were consistent with earlier findings of shorter survival after HIV diagnosis among IDUs compared with persons in other transmission categories [10]. Disparities between risk groups in the receipt of, or the adherence to, treatment might explain the observed differences in survival. Numerous studies have documented that the prescription of, and the adherence to, HAART is lower among IDUs than among persons who do not use drugs [14]–[16] because of IDUs' disadvantaged social situation, financial difficulties, and lack of family and social support [17]–[19]. Even in settings that provide free HIV treatment, low rates of utilization among eligible IDUs have been reported [20]. The use of HAART has been shown to be less common among active IDUs who do not have symptomatic diseases and thus have less contact with health care providers [5]. The strategies that have improved access and adherence to antiretroviral medications among HIV-infected IDUs include directly observed therapy, access to medical services without appointment, on-site pharmacists (at medical clinics), and improved access to addiction treatment [15], [21]. Information on treatment and type of health insurance, which might be related to receipt of treatment, was not available. In addition, deaths due to other causes (end-stage liver disease, bacterial infections), which are more common among IDUs than other risk groups, may play a role in the shorter survival of IDUs [22]. Future studies should assess whether the receipt of, or the adherence to, treatment can explain poorer survival of IDUs.

Shorter survival among IDUs may also be linked to late testing. A large proportion of IDUs learned of their HIV infection late in the course of disease, reducing optimal benefit from new therapeutic strategies, which remains a major public health issue. In an HIV testing study of persons at high risk for HIV infection, researchers found that the main reasons for not being tested were denial of HIV risk factors and fear of being HIV-positive [23]. Efforts to test HIV-infected people as early as possible should be made by increasing the awareness of HIV risk factors and decreasing the number of missed opportunities for testing. Addiction treatments and needle exchange programs are important venues to offer testing for HIV-positive IDUs. Understanding the psychological and social reasons for late testing among IDUs could help public health officials develop initiatives to reduce barriers to testing. CDC recommends routine testing in medical settings of all persons aged 13–64 years [24] to reduce the number of persons unaware of their HIV infection and diagnose HIV infection earlier in the disease process.

Similar to earlier analyses [11], our analysis revealed a lower probability of progression from HIV infection to AIDS within 1 year and within 3 years for female IDUs, compared with male IDUs. This finding may be explained by prenatal tests and gynecologic follow-up, which could increase the likelihood of HIV testing among female IDUs [25]. Persons, including IDUs, who receive a diagnosis of HIV infection at an older age are at increased risk of progressing from HIV diagnosis to AIDS or death within 1 and 3 years. Older age is a strong risk factor for disease progression because older persons have diminished immune function and more comorbidities [10], [26], [27]. In addition, our results show that older IDUs were more likely to receive a late diagnosis. As expected, CD4^+^ T-cell count at AIDS diagnosis was a strong determinant of survival [13], [28]. Guidelines for the use of antiretroviral medications in adults and adolescents infected with HIV type 1 recommend initiating treatment in patients with a history of an AIDS-defining illness or with a CD4^+^ T-cell count of <350 cells/µL [29]. Kohli et al. [22] reported that HIV-infected IDUs who began taking HAART at a CD4^+^ T-cell count of >200 cells/µL survived longer than those who began taking HAART at a CD4^+^ T-cell count of ≤200 cells/µL. Unfortunately, a substantial proportion of IDUs in the United States received a concurrent diagnosis of HIV and AIDS, and 38.2% of these persons had very low CD4^+^T-cell counts at HIV diagnosis (<50 cells/µL).

Our analyses are subject to several limitations. Although our data represent the largest set of available population-based data on HIV-infected persons, the data from 33 states may not be nationally representative, and it is not known whether the results can be extrapolated to the United States as a whole. However, all states and the District of Columbia have now implemented confidential name-based HIV reporting. The increased number of name-based reporting states will allow a more accurate description of the size of the population of HIV-infected IDUs and the determinants of disease severity. Risk factor information was missing for 27.1% of the persons with a diagnosis of HIV infection, so the data could not be included in our analysis. We also found that a large proportion of HIV-infected persons did not have a reported CD4^+^ T-cell count, which may reflect a lack of access to care or differences in reporting (e.g., state laws requiring laboratories to report). Finally, special research should be undertaken to better understand the reasons for late HIV testing (routine surveillance does not collect information about the reasons for late HIV testing).

In summary, we found that for large proportions of HIV-infected IDUs, diagnosis was made late in the disease process and HIV infection progressed to AIDS within 3 years. Also, survival among IDUs was shorter than among other HIV risk groups. These findings suggest that public health and medical services need to be improved to better target interventions for prolonging the lifespan of IDUs living with HIV infection, in addition to primary HIV prevention initiatives.
